# Verbal Autopsy: Reliability and Validity Estimates for Causes of Death in the Golestan Cohort Study in Iran

**DOI:** 10.1371/journal.pone.0011183

**Published:** 2010-06-17

**Authors:** Hooman Khademi, Arash Etemadi, Farin Kamangar, Mehdi Nouraie, Ramin Shakeri, Behrooz Abaie, Akram Pourshams, Mohammad Bagheri, Afshin Hooshyar, Farhad Islami, Christian C. Abnet, Paul Pharoah, Paul Brennan, Paolo Boffetta, Sanford M. Dawsey, Reza Malekzadeh

**Affiliations:** 1 Digestive Disease Research Center, Shariati Hospital, Tehran University of Medical Sciences Tehran, Iran; 2 Department of Epidemiology and Biostatistics, School of Public Health, Tehran University of Medical Sciences, Tehran, Iran; 3 Division of Cancer Epidemiology and Genetics, National Cancer Institute, National Institutes of Health, Bethesda, Maryland, United States of America; 4 Department of Public Health Analysis, School of Community Health and Policy, Morgan State University, Baltimore, Maryland, United States of America; 5 Department of Internal Medicine and Center for Sickle Cell Disease, Howard University, Washington, District of Columbia, United States of America; 6 International Agency for Research on Cancer, Lyon, France; 7 Department of Oncology and Department of Public Health and Primary Care, University of Cambridge, Cambridge, United Kingdom; 8 The Tisch Cancer Institute, Mount Sinai School of Medicine, New York, New York, United States of America; 9 International Prevention Research Institute, Lyon, France; Direccion General de Epidemiologia, Peru

## Abstract

**Background:**

Verbal autopsy (VA) is one method to obtain valid estimates of causes of death in the absence of valid medical records. We tested the reliability and validity of a VA questionnaire developed for a cohort study in Golestan Province in northeastern Iran.

**Method:**

A modified version of the WHO adult verbal autopsy was used to assess the cause of death in the first 219 Golestan Cohort Study (GCS) subjects who died. The GCS cause of death was determined by two internists who independently reviewed all available medical records. Two other internists (“reviewers”) independently reviewed only the VA answers and classified the cause of death into one of nine general categories; they repeated this evaluation one month later. The reliability of the VA was measured by calculating intra-reviewer and inter-reviewer kappa statistics. The validity of the VA was measured using the GCS cause of death as the gold standard.

**Results:**

VA showed both good validity (sensitivity, specificity, PPV, and NPV all above 0.81) and reliability (kappa>0.75) in determining the general cause of death independent of sex and place of residence. The overall multi-rater agreement across four reviews was 0.84 (95%CI: 0.78–0.89). The results for identifying specific cancer deaths were also promising, especially for upper GI cancers (kappa = 0.95). The multi-rater agreement in cancer subgroup was 0.93 (95%CI: 0.85–0.99).

**Conclusions:**

VA seems to have good reliability and validity for determining the cause of death in a large-scale adult follow up study in a predominantly rural area of a middle-income country.

## Introduction

Accurate information on overall and cause-specific mortality is essential to prioritize the activities of health systems and to efficiently invest scarce public health and medical care resources [1], [2], [3]. The availability of such information is also important for epidemiologic studies. The standard method to determine the cause of death is certification by an attending physician, based on valid medical documents, but this approach may yield unreliable results in many low- and middle-income countries, especially in rural and suburban areas. This is mainly due to the lack of infrastructure and the high cost of collecting the data, which limit access to information from diagnostic tests and post-mortem pathology services. Mortality data from these countries are therefore limited and potentially biased [3], [4], [5]. One relatively simple and low-cost alternative for determining a person's cause of death which is available in most low-resource countries is the so-called verbal autopsy (VA) [5], [6].

The VA methodology was first developed for investigating epidemics [7] and was later used for evaluations of outcomes of specific interventions [8], [9] and national mortality surveillance systems, principally in low-income countries such as India [10]. Several studies have shown that VA gives more valid causes of death than routine death certificate data in many developing countries [11], [12], [13], [14], [15]. In VA, a trained interviewer ascertains the symptoms, signs and events during the period leading up to death from family members or primary care givers of the deceased [6], [16]. This information is analyzed to derive a probable cause of death. The most commonly used method for analysis of the collected information is a “physician's review”, generally performed by more than one physician [6], [16]. Other methods, such as algorithms that can be applied by computer, have been tried but found to lack validity [17], [18], [19].

During 2004–2008, the Golestan Cohort Study (GCS) enrolled more than 50,000 adults in Golestan Province, in northeastern Iran [20], following a pilot study [21]. Golestan is a low-resource area of the country, and consequently, reliable clinical data are not available to determine the causes of death of the residents. Thus we have applied the VA method as a tool to identify the causes of death in the GCS. It is estimated that about 60% of the GCS participants will die at home, and some of them will not have any medical records accurately documenting their cause of death. VA represents an appealing approach to determine the cause of death in this group of subjects. However, it is necessary to validate the VA questionnaire in this adult population. The majority of VA validation studies have focused on neonatal and childhood mortality [12], [22], [23], [24], [25], [26], [27], [28], [29], [30], [31], [32], [33]. Only a few studies have investigated the validity of VA in adults [12], [25], [28], [32]. Although VA is prone to erroneous estimates of cause-specific mortality rates due to misclassification [34], several studies have demonstrated its ability in valid identification of the most common causes of death in many settings [12], [26], [27], [29], [32], [35], [36]. And even those who think VA is an imprecise tool for detecting the leading causes of death suggest that in the absence of other more reliable methods, VA may be useful as a secondary tool to determine causes of death in rural areas [6]. Our study is the first attempt to validate an adult VA questionnaire to be used in a longitudinal study in a medium income country.

## Materials and Methods

### The Golestan Cohort Study

The methods of the Golestan Cohort Study (GCS) have been previously described in detail [20]. In brief, 50,045 adult middle-aged individuals were enrolled in eastern Golestan Province, Iran between January 2004 and June 2008. Participants are actively followed through annual telephone contact to ascertain their vital and health status. If a participant cannot be reached, family members, friends, or local health workers are contacted. Moreover, local health workers in rural areas, called “Behvarz”, are contacted monthly to inquire about any possible outcomes, including death. In the event of death, the follow-up team performs two main tasks in parallel. First, a trained general practitioner goes to the homes of the family members or primary care givers of the deceased and conducts a VA interview. Second, the team determines which physicians or hospitals were visited by the decedent and obtains all medical documents (charts, X-rays, pathology reports, etc) that could be used to identify the cause of death. These documents may be available in Golestan or in neighboring provinces.

The GCS follow-up team uses the adult VA questionnaire originally developed by World Health Organization (WHO) and the International Network of field sites with continuous Demographic Evaluation of Populations and Their Health in developing countries (INDEPTH) [37], [38], with some modifications to adapt to the local situation in Golestan. We tailored the standard VA questionnaire based on cultural background and education of study population. We made special attention to the most common disease and causes of death in the study area. We added some disease-oriented questions for specific diseases (cardiovascular, stroke, cancer (esophageal and gastric), diabetes, hypertension, tuberculosis and asthma) to collect more information by VA. Since we have already collected the data of life style and personal habits of the study participants at the enrollment phase of GCS, we excluded this part of VA questionnaire to save time. Local terms for some signs/symptoms such as “dysphagia” were applied when we translated the VA questionnaire to Farsi.

After the VA interview and medical document search are completed, the results are given to two internists to ascertain the cause of death. The two internists who review the VA and other documents are unaware of each other's diagnosis. When they disagree on the cause of death, a third senior internist reviews the VA, the available documents, and also the diagnoses of the first two internists and makes the final decision. All causes of death are coded according to the core three digit codes of the International Classification of Diseases, Tenth Revision (ICD-10) [39]. The cause of death obtained by this method was considered as the gold standard for the current validation study. Seventy cases (32%) had no medical documents, so in these cases the VA-based diagnoses confirmed by the above method were used as the gold standard.

### Validation study

This validation study was conducted on all 219 deaths reported in GCS participants by the end of January 2005. Copies of all 219 completed VA questionnaires were given to two trained internists, henceforth referred to as the “reviewers”, who were different individuals from the internists who made the first GCS cause of death determinations. The reviewers studied the completed VA questionnaires independently, and made their decisions on the cause of death based on the VA questionnaire alone, without having any other medical documents. In order to get an estimate of within-reviewer reliability of the VA diagnoses, the same two reviewers were asked to review the VA's a second time one month later, without the knowledge that this was a repeat review.

For the purpose of this study the causes of death were categorized into one of nine major categories. To estimate the reliability, kappa statistics were calculated for the agreement between the reviewers' diagnoses. Both within-reviewer reliability (comparing the first and second diagnoses of the same reviewer) and between-reviewer reliability (comparing the diagnoses made by the two reviewers) were calculated. Multi-rater agreement was calculated and its confidence interval was calculated using bootstrap technique. To estimate validity, the VA diagnoses made by the two reviewers were compared to the gold standard diagnoses and the sensitivity, specificity, positive predictive value (PPV), negative predictive value (NPV) and kappa statistics were calculated for each reviewer-gold standard comparison. As a sensitivity analysis, the VA validity was once calculated in the subgroup of 149 cases (68%) who had both VA and medical documents available for the gold standard cause of death determinations, and then in all 219 cases.

All study participants had signed written informed consent at enrollment phase of GCS and ethical approval for the present study was obtained from the ethics committee of Digestive Disease Research Center of Tehran University of Medical Sciences.

## Results

Of the 219 deceased participants, 133 (60.7%) were male and 86 (39.3%) were female. The mean age (±standard deviation) at death was 64.4±10.7 years. Among the deceased, 91 (41.6%) were urban and 128 (58.4%) were rural dwellers. In most cases (85%), the respondent lived with the deceased at the time of death. Of the 219 deaths in the validation study, 70 (32%) had no medical record other than the completed VA.

Table 1 presents the major causes of deaths according to the gold standard diagnoses, among the total study population and the subgroup of 149 subjects (68%) who had both VA and medical documents available. Ischemic heart disease, cancers, cerebrovascular events, and transportation accidents were the most common causes of death, respectively, and were responsible for, approximately 80% of deaths.

**Table 1:** The distribution of causes of death in all 219 deceased and the subset of 149 deceased with supporting medical documents.

No.	Cause of Death	Complete set Number (%)	Subset Number (%)
**1**	**Ischemic Heart Diseases (IHD)**	81 (37%)	50 (34%)
**2**	**Cancers**	49 (22%)	41 (27%)
**3**	**Cerebrovascular diseases (CVA)**	33 (15%)	19 (13%)
**4**	**Transport Accidents**	16 (7.4%)	7 (4.7%)
**5**	**Renal diseases**	8 (3.8%)	7 (4.7%)
**6**	**Pulmonary diseases**	6 (2.8%)	5 (3.3%)
**7**	**Liver diseases**	5 (2.4%)	4 (2.6%)
**8**	**Unknown**	14 (6.4%)	9 (6.0%)
**9**	**Other**	7 (3.2%)	7 (4.7%)
	**Total**	**219 (100%)**	**149 (100%)**

Table 2 shows the results of kappa statistics for the within and between reviewer diagnoses and the comparison of the VA diagnoses with the gold standard, based on the 149 deaths with documentation available.

**Table 2:** Kappa (κ) statistics for reliability and validity testing of VA interview in 149 documented deaths.

Comparison	κ statistic in Total	κ statistic in Males	κ statistic in Females	κ statistic in Urban	κ statistic in Rural
**A1 vs A2**	0.91	0.87	0.98	0.93	0.89
**B1 vs B2**	0.89	0.88	0.91	0.92	0.87
**A1 vs B1**	0.81	0.81	0.81	0.73	0.86
**A2 vs B2**	0.81	0.79	0.84	0.83	0.79
**A1 vs B2**	0.80	0.80	0.81	0.79	0.81
**A2 vs B1**	0.82	0.81	0.83	0.77	0.85
**A1 vs GS**	0.79	0.77	0.84	0.78	0.86
**A2 vs GS**	0.86	0.86	0.86	085	0.87
**B1 vs GS**	0.82	0.83	0.79	0.75	0.87
**B2 vs GS**	0.82	0.84	0.79	0.79	0.84

A1: reviewer A first diagnosis, A2: reviewer A second diagnosis, B1: reviewer B first diagnosis, B2: reviewer B second diagnosis, GS: gold standard.

The overall multi-rater agreement across four reviews was 0.84 (95%CI: 0.78–0.89). Most pairwise kappas were higher than 0.80, indicating good within-reviewer and between-reviewer reliability, the within-reviewer reliability being somewhat better than between-reviewer reliability. Agreement between each reviewer and the gold standard was also good (kappa>0.75).

Sensitivity, specificity, and predictive values for the four most common causes of death are presented in Table 3. To analyze sensitivity, these were calculated for the A1 review which had the lowest agreement with the gold standard and then for the one with the highest agreement (A2). All estimates were higher than 0.81 which indicate good validity. As expected transportation accidents had the highest validity.

**Table 3:** Validation results for VA reviews in diagnosing cause of death for 4 selected causes in 149 documented deaths.

Cause	GS deaths	VA deaths	Sensitivity (95% CI)	PPV (95% CI)	Specificity (95% CI)	NPV (95%CI)
**A1**						
**IHD**	50	52	90.0 (81.5–98.5)	86.5 (77.1–96.0)	92.9 (87.8–98.1)	94.8 (90.4–99.3)
**Cancer**	41	42	97.6 (92.7–100)	95.2 (88.7–100)	98.1 (95.5–100)	99.1 (97.2–100)
**Cerebrovascular diseases**	19	22	94.7 (84.5–100)	81.8 (65.4–98.3)	96.9 (93.9–99.9)	99.2 (97.6–100)
**Transport accidents**	7	7	100	100	100	100
**A2**						
**IHD**	50	57	98.0 (94.0–100)	86.0 (76.8–95.2)	91.9 (86.4–97.4)	98.9 (96.7–100)
**Cancer**	41	43	100	95.3 (88.9–100)	98.1 (95.5–100)	100
**Cerebrovascular diseases**	33	32	89.5 (75.4–100)	89.5 (75.4–100)	98.5 (96.3–100)	98.5 (96.3–100)
**Transport accidents**	7	7	100	100	100	100

A1: review with the lowest kappa according to table 2 (Reviewer A's first review).

A2: review with the highest kappa according to table 2 (Reviewer A's second review).

Since the main goal for Golestan Cohort Study is to study the causes of upper GI cancers in particular and other cancers in general, we also tested the validity of VA for different types of cancer. Of 41 cancer deaths (in 149 deaths), 13 were due to esophageal cancer, the others being due to gastric cancer (n = 5) liver cancer (4), lymphoma (4), lung cancer (3) leukemia (3), breast cancer (2) and other cancers (7). In the comparison between A1, A2, B1 and B2 review results versus GS, the kappas were 0.82, 0.85, 0.78, and 0.85, respectively for all types. The multi-rater agreement for four reviews was 0.93 (95%CI: 0.85–0.99). In addition, the validity of VA in detecting upper GI cancers was 0.95 for all reviews.

To check the differences between documented and non-documented causes of death, we did the same analysis on 219 VA (Tables 4, 5). The numbers are comparable to those in Table 2, 3.

**Table 4:** Kappa statistics for reliability and validity testing of VA interview in 219 deaths.

Comparison	κ statistic in Total	κ statistic in Males	κ statistic in Females	κ statistic in Urban	κ statistic in Rural
**A1 vs A2**	0.89	0.86	0.99	0.90	0.89
**B1 vs B2**	0.89	0.84	0.91	0.92	0.87
**A1 vs B1**	0.81	0.78	0.86	0.75	0.86
**A2 vs B2**	0.84	0.82	0.86	0.85	0.83
**A1 vs B2**	0.80	0.77	0.83	0.79	0.81
**A2 vs B1**	0.84	0.83	0.86	0.78	0.88
**A1 vs GS**	0.80	0.76	0.87	0.80	0.80
**A2 vs GS**	0.86	0.86	0.86	0.83	0.89
**B1 vs GS**	0.84	0.83	0.85	0.79	0.88
**B2 vs GS**	0.84	0.85	0.82	0.79	0.88

A1: reviewer A first diagnosis, A2: reviewer A second diagnosis, B1: reviewer B first diagnosis, B2: reviewer B second diagnosis, GS: gold standard.

**Table 5:** Validation characteristics of VA interviews in diagnosing cause of death for 4 selected causes in 219 deaths.

Cause	GS deaths	VA deaths	Sensitivity (95% CI)	PPV (95% CI)	Specificity (95% CI)	NPV (95%CI)
**A1**						
**IHD**	81	88	93.8 (88.5–99.2)	86.4 (79.0–93.7)	91.3 (86.5–96.1)	96.2 (92.8–99.5)
**Cancer**	49	53	98.0 (93.9–100)	90.6 (82.5–98.6)	97.1 (94.5–99.6)	99.4 (98.2–100)
**Cerebrovascular diseases**	33	34	90.9 (80.9–100)	88.2 (77.2–99.3)	97.8 (95.7–100)	98.4 (96.5–100)
**Transport accidents**	16	15	93.7 (93.0–94.5)	100	100	99.5 (98.5–100)
**A2**						
**IHD**	81	94	98.8 (96.3–100)	85.1 (77.8–92.4)	89.8 (84.7–95.0)	99.2 (97.6–100)
**Cancer**	49	51	100	96.1 (90.6–100)	98.8 (97.2–100)	100
**Cerebrovascular diseases**	33	32	90.9 (80.9–100)	93.7 (85.2–100)	98.9 (97.4–100)	98.4 (96.5–100)
**Transport accidents**	16	16	100	100	100	100

A1: review with the lowest kappa according to table 2 (Reviewer A's first review).

A2: review with the highest kappa according to table 2 (Reviewer A's second review).

## Discussion

Verbal autopsy seems to be a reliable and valid supplemental method to assess causes of death in the Golestan Cohort Study with comparable results in men and women and for patients from both rural and urban areas. One major reason for the usefulness of VA in the GCS may be the appropriate modifications made in the adult questionnaire prepared by WHO and the INDEPTH [37], [38] to adapt it to the local setting. Our results are consistent with those of most previous studies, showing that the VA is a reasonably valid tool to ascertain causes of death [12], [26], [27], [29], [32], [35], [36]. Some other studies are less supportive of the VA [34], and some even suggest that VA is not a very precise tool for detecting the leading cause of death among adults [6]. The reason for inconsistency in results of VA validation studies may be that VA is a developing method itself [40]. Thus, there are several variations of VA methodology and questionnaires, and some studies have not use the ICD coding system for their cause of death diagnoses. WHO has recently published instructions to improve the quality and standards for use of this method [41].

We used the VA method in the GCS, which is the first large-scale prospective population-based cohort study of cancer in the Middle East, to improve the accuracy of diagnosing the causes of death of cohort members. The majority of families in the rural area of Golestan Province prefer their family members to die at home after a diagnosis of end-stage cancer. About 60% of the decedents in the current study died at home, and only half of these had a prior hospital-based diagnosis; for the other half, the VA seems to be a promising approach to identify at least a general cause of death.

The kappa statistics obtained in the current study show that VA generates highly reliable results, at least among the 9 major categories of causes of death used in our study. Our results showed both high within–reviewer and between-reviewer reliability. We also found this method to be valid, with high sensitivity and specificity when compared to the gold standard diagnoses. Our results for making the diagnosis of different cancer subtypes also seem promising. This is especially true for upper gastrointestinal (UGI) cancers, the main focus of the GCS. Dysphagia, the main symptom of esophageal cancer, is very characteristic of this disease, and the availability of at least 10 UGI endoscopy clinics in the region, three providing free-of-charge endoscopy services to the GCS subjects, has made it possible to have accurate histologic diagnoses for almost all UGI cancers.

There are of course several caveats and methodological considerations related to this method. The gold standard was set by a combination of diagnoses made by two internists, or a third internist when the first two internists did not concur. These physicians used both VA and other clinical documents to adjudicate the results, but in 32% of the cases both the original internists and the reviewers in our study had only the VA answers to review. This lack of additional clinical documents in a third of the cases might raise concern that our validity estimates were falsely elevated, but this does not seem to have been the case, since these estimates were essentially identical in the full group of cases and in the subgroup which had additional clinical documents. On the other hand, our results may underestimate the potential sensitivity and specificity of the VA method because they tested the diagnosis made by each reviewer separately. In the actual GCS, at least two internists and perhaps a third one decides on the final diagnosis, and therefore the results may be more accurate than the judgment of just one physician.

In conclusion, our results suggest good reliability and good validity of verbal autopsy in determining the causes of death in a large-scale adult cohort study in a predominantly rural area in a developing country. These results add to the current literature on the use of VA for cohort studies in adult populations.
